# Estimation of the commercial height of trees with laser meter: a viable alternative for forest management in the Brazilian Amazon

**DOI:** 10.1002/ece3.4102

**Published:** 2019-09-30

**Authors:** Agust Sales, Marco Antonio Siviero, Paulo Cezar Gomes Pereira, Sabrina Benmuyal Vieira, Ghaby Alves Berberian, Bárbara Maia Miranda

**Affiliations:** ^1^ Department of Forestry Federal University of Viçosa Viçosa Brazil; ^2^ The Arboris Group Dom Eliseu Brazil; ^3^ Department of Technology and Natural Resources University of the State of Pará Belém Brazil; ^4^ Department of Environmental Science Federal University of Pará Belém Brazil; ^5^ Municipal Secretariat of Environment Dom Eliseu Brazil

**Keywords:** dendrometry, forest measurement, tree stature, trigonometric principles, tropical forest

## Abstract

Commercial height of the tree is a key variable for estimating the wood stock in tropical forests managed for timber production purposes. Most available measurement devices suffer limitations in this type of forest, promoting low precision measurements with high variation errors. The laser meter device appears as a viable alternative, as in addition to using trigonometric principles, it is not necessary that the device is close to the eyes of the meter to carry out the measurement. The device can be used to measure commercial height of trees on flat or sloping terrain, at different distances from the tree. However, there are no studies evaluating the precision of this device. The objective of this study was to determine the precision of the laser meter method for estimating the commercial height of trees, as compared to the actual measurement in a tropical forest in the Brazilian Amazon. Measurements were made on 300 trees with commercial height between 7 and 14 m. Actual commercial heights were measured with graduated ruler. Applied tests were paired *t* test, graphical analysis of residuals and calculations of bias statistics, mean absolute deviation, standard deviation of differences, and coefficient of determination (*R* ^2^). Paired *t* test indicated that the mean of the heights measured by the laser meter is statistically equal to that of the graduated ruler. Measurements with laser meter did not show bias and had mean error of 0.0745. The standard deviation of the differences indicated dispersion of errors of 0.97, equal to that shown in the graduated rule. Laser meter presents an alternative method for estimating the commercial height of trees in tropical forest in the Brazilian Amazon. There was no tendency to underestimate or overestimate the commercial heights of trees. Use of the laser meter is potentially of use for measuring the commercial height of trees in tropical forests.

## Graphical abstract

A new method and device to measure commercial height of trees in natural forests is proposed. The method has, besides practicality, good precision, and low cost, a great differential: The technician does not need to use the device close to the eyes. The technician observes the laser beam in the tree trunk and remains in the same position to realize the three measures necessary to obtain the commercial height. The device can be used to measure commercial height of trees on flat or sloping terrain from any distance from the tree.

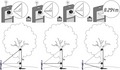

## 1 INTRODUCTION

The management of tropical forests for timber production purposes in the Brazilian Amazon is carried out with the approval of the sustainable forest management plan (SFMP) by the competent environmental agencies. Among the requirements for SFMP approval, it is required the quantification of the wood stock of the feasible trees for harvest (CONAMA, 2009). The wood stock is quantified by estimates generated from volume equations specifically for SFMP, using the variables diameter at breast height and commercial height (usually the distance between tree base and beginning of the tree crown; Burkhart & Tomé, 2012).

Measuring the commercial height of trees is a difficult in tropical forests when compared to planted forests (Avery & Burkhart, 2015). This process becomes complex due to the density, diversity, and other obstacles inherent in the tropical forest environment (Clark, Olivas, Oberbauer, Clark, & Ryan, 2008; Primack & Corlett, 2011). For tree height estimates, there are varied types of equipment that are used internationally, called hypsometer, and classified into two categories, according to their construction principle: geometric principle, based on the relation between triangles, such as the hypsometer of Christen; and the trigonometric principle, which is based on the relationship between angles and distances.

Most commonly used devices for standing trees height estimation are based on trigonometric principles, and it is necessary for the technician to maintain the known distance from the tree (Loetsch, Zohrer, & Haller, 1973). In some equipment, such as digital clinometer, the distance is predetermined. Laser hypsometers or ultrasound waves allow to measure at different distances, and generally, the recommended distance from the technician to the tree is the height to be measured (Campos & Leite, 2017). On the other hand, in other cases, the technician needs to be close to the tree so as not to lose sight of the crown. In this case, increasing the angle reduces accuracy (Larjavaara & Muller‐Landau, 2013; da Silva, Curto, Soares, & Piassi, 2012; da Silva, de Oliveira, Martinelli de Souza, Boechat Soares, & Lemoss, 2012).

The relatively low precision (errors ± 40%) of height measuring devices in tropical forests encourages the use of conventional direct measurement (real height) methods, such as the use of graduated or telescopic rulers, which results in expensive and costly (da Silva, de Oliveira, et al., 2012; Kearsley et al., 2017). It is important to emphasize that these factors motivate some companies to perform the visual estimation commercial height of trees, with or without technician training, generating under‐ or overestimation.

In an attempt to solve this difficulty and obtain commercial height data in tropical forests with higher precision, laser distance meter arises as an alternative. This device is also based on trigonometric principles; however, the great difference is that it is not necessary that the device is close to the eyes of the technician to carry out the measurement. Measurement is made with the device at any distance from the eyes as long as the technician remains in the same position to make three measurements necessary to obtain the commercial height.

**Figure 1:**
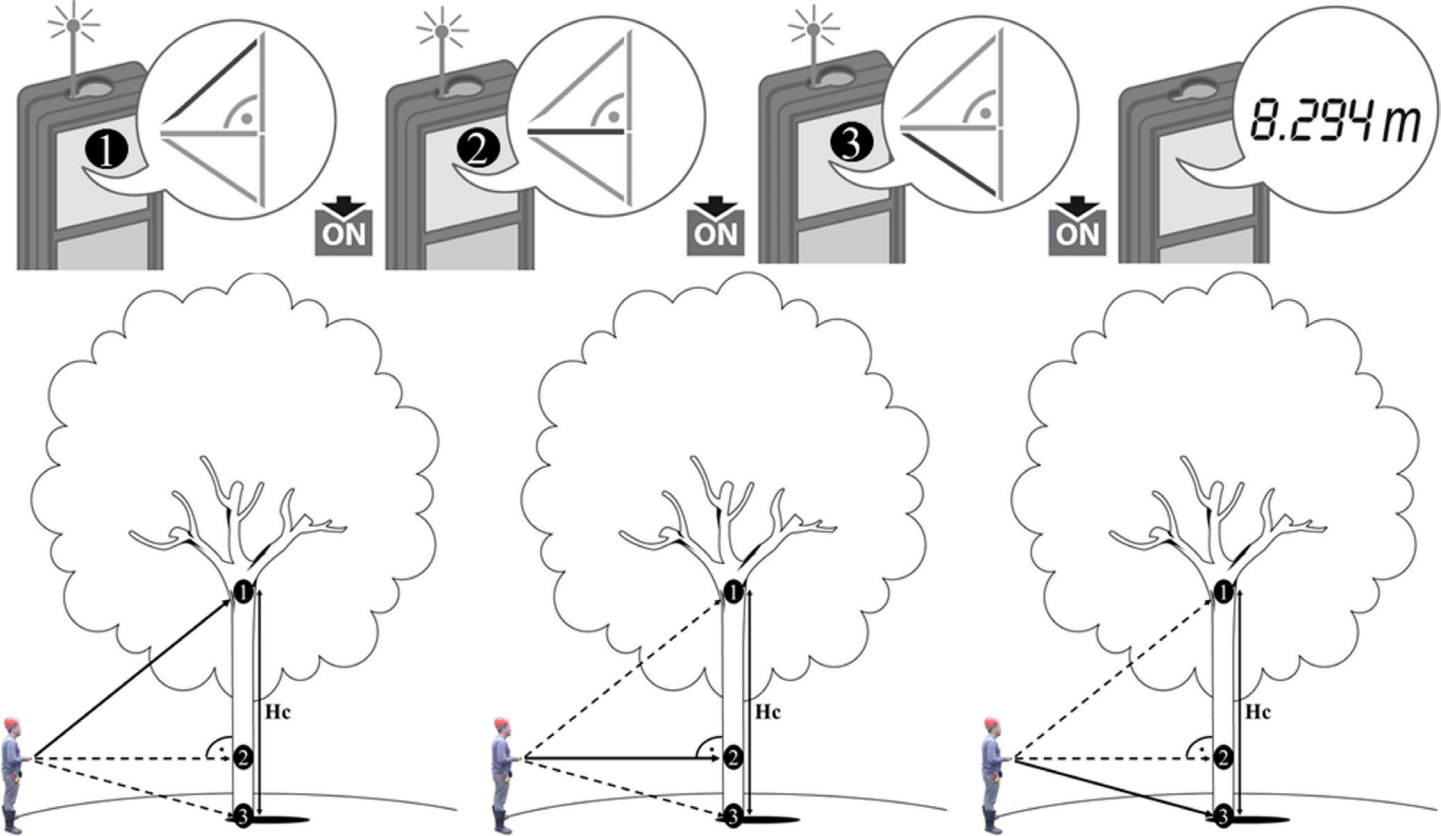
Procedure for obtaining commercial height. The distances 1, 2, and 3 are measured as indicated on the display. After measurement 3, the commercial height of the tree, in meters, is displayed on the device screen

Laser meter is based on the Pythagorean theorem, which expression can be applied to any triangle of 90° angle, using the proof of similar triangles (Loomis, 1968). Use of the device is very simple, the technician measures the distance in three points, perpendicular to the operator, in tree trunk (upper, rectangular, and bottom), one at a time, in the order determined on the device's screen. The height is calculated automatically on the device's screen (Figure 1). The device can be used to measure commercial height of trees on flat or sloping terrain from any distance from the tree and makes it possible to carry out permanent measurements, thus achieving maximum and minimum values.

Researches on the use of this apparatus are nonexistent; thus, it is necessary to determine its accuracy in the measurement of the commercial height of trees in tropical forests. Due to importance and difficulties evidenced for the height estimation of trees in tropical forests, the objective of this work was to determine the precision of the laser meter method for the estimation of the commercial height of trees, as compared to the direct measurement with graduated ruler, in a tropical forest in the Brazilian Amazon.

## 2 MATERIALS AND METHODS

### 2.1 Study site

The experiment was carried out in a forest of Fazenda Gênesis 4°01′56″S and 47º36′19″W in 481 ha, municipality of Dom Eliseu, southeast of Pará state, Brazil. Fazenda Gênesis belongs to the Arboris Group, a business group that works with agriculture and forestry in the Amazon, including wood production from planted and native forests. In Fazenda Gênesis, the vegetation is dominated by submontane dense ombrophilous forest (IBGE, 2004). The average temperature is 26.5°C with annual precipitation of 2,200 mm and rainy season from January to May. Relative air humidity indicates an annual average of 85%. The average altitude in the study area is 180 m, and the most common soil type is Yellow Oxisol (EMBRAPA, 2013; SUDAM, 1993; Veloso, Rangel‐Filho, & Lima, 1991).

### 2.2 Materials used

Materials used were a graduated ruler and a laser meter. Graduated ruler has a length of 14 m when fully opened and 2 m fully closed, weighing around 8 kg. The device used was the Makita Laser Meter LD050P which can cost up to 1/10 of the value of the most used devices for measuring tree height. It has dimensional advantages, as it is only 11.6 cm in its largest dimension, weighs approximately 100 g (with battery), and has a measuring range of 50 m with a precision of +2 mm, besides being resistant to falling and splashing water and shows the readings directly in the digital format.

The laser meter device calculates tree height by simply measure distance of three points on the tree trunk. The first point is the commercial height to be measured, second point is in the rectangular direction for forming a right triangle, and the third point is at the base of the tree. Formation of two rectangular triangles allows the commercial height to be obtained at any distance from the tree and on sloping terrain.

### 2.3 Data collection and analysis

Commercial heights were measured, between 7 and 14 m, being the maximum range offered by the graduated rule, in 300 trees randomly selected. Measurements made with the graduated ruler and with the laser meter were obtained by the same technician, to carry out the control and to avoid the influence of the technician on the collected data. Statistical analyses were performed using the software RStudio^®^ and Microsoft Office Excel^®^ 2013.

Height frequency histogram was established with the number of classes determined by the Sturges method (1926). Paired *t* test (α = 0.05) was applied in order to compare the commercial heights obtained by the laser meter in relation to those obtained by the graduated ruler. The hypotheses of the test were as follows:

$$\text{Null hypothesis}\;(H_{0}):\mu l=\mu r$$

$$\text{Alternative hypothesis}\;(H_{a}):\mu l\neq \mu r$$

where μ*l* = commercial heights averages measured with laser meter and μ*r* = commercial heights averages measured with graduated ruler.

Furthermore, the residuals from the commercial heights obtained with laser meter were calculated as follows:

$$\text{Error}\;\left(\%\right)=\frac{Yi-\hat{Y}i}{Y}100$$

where $\hat{Y}$ **= heights measured with laser meter and *Y *= heights measured with graduated ruler.

Additional statistical tests were applied to complement the graphical analysis of the residuals: bias, mean absolute deviation (MAD), and standard deviation of differences (*S*_d_). The bias shows the existence of some tendency among the residues, the MAD reflects the amplitude between the residues, and the *S*_d_ expresses homogeneity of the residuals. Bias:

$$\text{Bias}=\frac{\left(\sum_{i=1}^{n}Yi-\sum_{i=1}^{n}\hat{Y}i\right)}{n}$$

1. Mean absolute deviation:
  $$\text{MAD}=\frac{\sum_{i=1}^{n}\left|Yi-\hat{Y}i\right|}{n}$$
2. Standard deviation of differences:
  $$S_{d}=\sqrt{\frac{\sum_{i}^{n}di^{2}-\frac{{\left(\sum_{i}^{n}di\right)}^{2}}{n}}{n-1}}$$
3. Coefficient of determination:
  $$R^{2}=1-\frac{\sum {\left(Y-\hat{Y}\right)}^{2}}{\sum {\left(Y-\overset{¯}{Y}\right)}^{2}}$$
4. Sturges’ formula (1926):
  $$N_{c}=1+\left(3,333\times \log(n)\right)$$

where *Yi* = heights measured with graduated ruler, $\hat{Y}i$ = heights measured with laser meter, *n* = number of observations, $di=(Yi-\hat{Y}i)$, $\overset{¯}{Y}$ = heights averages measured with graduated ruler, and *N*_c_ = number of classes.

## 3 RESULTS

**Figure 2:**
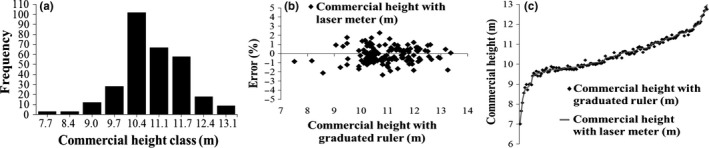
Frequency distribution of trees with commercial height greater than 7 m and less than 14 m (a), distribution of residuals of height, in percentage, for the laser meter method (b), and line estimate of the heights measured with laser meter (c)

Sturges’ method resulted in nine heights classes with a class interval of 0.7 m. Frequency distribution of the heights was set up in a normal distribution to apply the *t* test paired, in order to compare the heights measured by the graduated ruler and laser meter (Figure 2).

Paired *t* test performed did not reject *H*_0_, and the mean of the heights measured with laser meter is statistically equal to that of the graduated ruler, with *p*‐value > .05. The *t*_calc_ is smaller than the critical value (0.05; 299) of 1.960 (Table 1).

**Table 1:** Statistical method of analysis and performance of methods laser meter and graduated ruler

Method	Commercial height (m)				Statistics				
	Min	Max	Mean	Standard deviation	*T*_calc_	Bias	MAD	*S*_d_	*R* ^2^
Graduated ruler	7.0	12.9	10.45	0.97	0.1930^ns^	−0.0173	0.0745	0.0872	.99
Laser meter	7.1	13.0	10.47	0.97					

ns, Nonsignificant at 5% probability.

Commercial heights measured with laser meter showed no bias and had a mean error (MAD) of 0.0745, indicating homogeneity. The standard deviation of the differences indicated dispersion of errors of 0.97, equal to that shown in the graduated rule (Table 1).

The errors plotted in graphs to facilitate the visualization of its distribution show that they varied between ±2.5% and that there are no tendencies. Homogeneity and nonbias observable between the methods are shown with the estimated line drawn from the commercial heights measured with the laser meter simultaneously to the cloud of points represented by the heights measured by the graduated ruler (Figure 2).

## 4 DISCUSSION

Acceptance of *H*_0_ provides evidence that there is no statistical difference between measurement methods in this case, for these trees. Paired *t* test allows the comparison to be more practical and easy to understand, as other factors may influence the result in any two samples. This test is extremely useful for comparing the same sample in two different methods (Rosner, 1995).

The bias, when negative, indicates a tendency to overestimate, and positive, underestimate. Bias and mean absolute deviation zero indicate a lower trend and greater homogeneity, respectively, and the lower the standard deviation of differences, the smaller the amplitude of the residuals (Snowdon, 1992).

The method of commercial height measurement with laser meter shows relatively minor values of trends and amplitude and variation of errors (Table 1, Figure 2), when compared to other studies in which researchers compared measures of portable instruments based on trigonometric principles with actual heights obtained by conventional methods: David, Araújo, Pelissari, Miranda, and Ebling (2012) compared total heights measured with Haglof digital clinometer with real heights measured with telescopic ruler, obtaining MAD of 0.763 m, *S*_d_ in average of 1.0 m and errors around ±25%. da Silva, de Oliveira, et al. (2012) conducted two studies: one in an eucalyptus plantation and the other in a natural forest (da Silva, Curto, et al., 2012). In the plantation, the mechanical clinometers presented better performance than the electronic ones, and the bias was significant when the distance to the tree was much smaller than the height of the tree. In the natural forest, the random errors were larger, while the bias remained unimportant.

The comparison of electronic devices in measurements of height and diameter for purposes of forest inventory made by Couto and Bastos (1980) indicated that the highest percentage errors were obtained in the lowest real heights (trees <10 m), with variation of ±17.11%. There was, among the different situations evaluated, the interference of the technician to the tree in the estimation.

Difficulty in minimizing errors is related to the specifications of most forestry measuring devices that recommend the technician to measure the tree at a distance equivalent to the height to be measured (Curto, Silva, Soares, Martins, & David, 2013). However, due to the great variation of the canopy stratum and heterogeneity of spacings in tropical forests, the synchronous visualization of the base and the canopy of the tree is difficult. The difficulty in visualizing the tree forces the technician to measure at smaller distances. This increases the slope sensitivity of the device, generating greater variation in height reading and, consequently, increasing erroneous estimates (Campos & Leite, 2017; Freitas & Wichert, 1998; Larjavaara & Muller‐Landau, 2013).

None of the cited studies included a comparison of commercial height measurements with a laser meter. The laser meter method proposed in this work does not present a slope sensitivity; thus, it produces less reading variation when compared to other devices, reducing the risks of error in estimates. It is likely that the relationship between commercial heights measured with laser meter and graded ruler will vary little between forests and potentially between tree species and seasons, depending on the structure of the forest, the state of the canopy at the time of measurement, etc.

The relationship between commercial heights may also vary between the models of the laser meter, as it is likely to depend on the power of the laser beam (and therefore of the laser visualization when it reaches the shaft of the tree) and the proportion of reflection based on which distance is calculated and detector settings in general, including technical specifications that may be proprietary and which may change over time as new models replace older ones. It is possible that laser‐specific correction functions are developed for forest types, but this would require a lot of additional research in this field.

## 5 CONCLUSIONS AND RECOMMENDATIONS

The laser meter has been shown to be at least as good as “standard” methods for estimating the commercial height of trees in tropical forest in the Brazilian Amazon. There was no tendency to underestimate or overestimate the commercial heights of trees.

The use of the laser meter is only suggested here for measuring the commercial height of trees in tropical forests, not the total height, or for forests with grester. It is not recommended for measuring total height of trees and no measurements on forests planted with greater spacing between trees. In measuring total height, the leaf area of the tree can affect the laser reflectance, causing an error in the measurement of the device. In forests planted with trees at spacings larger, there is a high incidence of light in the tree trunk, affecting the visualization of the laser.

Future studies should evaluate this type of apparatus in other forest conditions that differ in structure and with other instruments that differ in the specifications to develop a better basis for estimating the actual commercial heights of these measurements.

It would be particularly interesting to evaluate the accuracy of the laser meter in trees of commercial heights greater than 14 m. This could be easily accomplished by comparing with true commercial heights measured on trees after harvest.

## CONFLICT OF INTEREST

None declared.

## AUTHORS’ CONTRIBUTIONS

AS and MAS conceived the ideas and designed the study. AS collected data. AS and PCGP conducted analysis of data and perfected the method. AS, MAS, and PCGP wrote the article. All authors contributed critically to the drafts and gave final approval for publication.
